# ARF6-Dependent Regulation of P2Y Receptor Traffic and Function in Human Platelets

**DOI:** 10.1371/journal.pone.0043532

**Published:** 2012-08-16

**Authors:** Venkateswarlu Kanamarlapudi, Sian E. Owens, Keya Saha, Robert J. Pope, Stuart J. Mundell

**Affiliations:** 1 Institute of Life Science, College of Medicine, Swansea University, Swansea, United Kingdom; 2 School of Physiology and Pharmacology, Medical Sciences Building, University of Bristol, Bristol, United Kingdom; Heart Center Munich, Germany

## Abstract

Adenosine diphosphate (ADP) is a critical regulator of platelet activation, mediating its actions through two G protein-coupled receptors, the P2Y_1_ and P2Y_12_ purinoceptors. Recently, we demonstrated that P2Y_1_ and P2Y_12_ purinoceptor activities are rapidly and reversibly modulated in human platelets, revealing that the underlying mechanism requires receptor internalization and subsequent trafficking as an essential part of this process. In this study we investigated the role of the small GTP-binding protein ADP ribosylation factor 6 (ARF6) in the internalization and function of P2Y_1_ and P2Y_12_ purinoceptors in human platelets. ARF6 has been implicated in the internalization of a number of GPCRs, although its precise molecular mechanism in this process remains unclear. In this study we show that activation of either P2Y_1_ or P2Y_12_ purinoceptors can stimulate ARF6 activity. Further blockade of ARF6 function either in cell lines or human platelets blocks P2Y purinoceptor internalization. This blockade of receptor internalization attenuates receptor resensitization. Furthermore, we demonstrate that Nm23-H1, a nucleoside diphosphate (NDP) kinase regulated by ARF6 which facilitates dynamin-dependent fission of coated vesicles during endocytosis, is also required for P2Y purinoceptor internalization. These data describe a novel function of ARF6 in the internalization of P2Y purinoceptors and demonstrate the integral importance of this small GTPase upon platelet ADP receptor function.

## Introduction

Adenosine diphosphate (ADP) plays a key role in platelet function. Following its secretion from platelet-dense granules ADP amplifies the platelet responses induced by other platelet agonists and stabilizes platelet aggregates. ADP activates two platelet G protein-coupled receptors (GPCRs), P2Y_1_ and P2Y_12_, which couple respectively to G_q_ and G_i_-mediated pathways, and synergise to induce full platelet aggregation responses to ADP [1], [2]. Interaction of ADP with P2Y_1_ leads to mobilization of intracellular calcium and activation of Rho kinase, resulting in platelet shape change and an initial wave of rapidly reversible aggregation. In contrast, ADP stimulation of P2Y_12_ is associated with adenylyl cyclase (AC) inhibition and PI3-kinase activation, resulting in sustained aggregation in synergy with the P2Y_1_ receptor. Activation of both receptors is required for a full aggregation response to ADP [3].

Recent work from our laboratory has shown that P2Y receptor responsiveness is rapidly and reversibly modulated in human platelets [4]. Upon prolonged exposure to agonist, the responsiveness of both P2Y_1_ and P2Y_12_ purinoceptors, decreases in human platelets through different kinase-dependent mechanisms [5]. More recently, we have demonstrated that clathrin-dependent agonist-induced internalisation, receptor dephosphorylation and subsequent receptor recycling is required for the rapid resensitisation of P2Y receptor function in human platelets [4]. Given the importance of these receptors in both physiological and pathophysiological context, understanding of how P2Y receptor function is regulated is essential for development of new antiplatelet agents [6] both to improve existing therapies and to provide novel therapeutic targets.

The ADP-ribosylation factor (ARF) family of small GTPases has been implicated in the regulation of vesicle trafficking [7]. These proteins regulate trafficking by shuttling between an active GTP-bound form and an inactive GDP-bound form. Of the six mammalian ARF isoforms (ARFs 1–6), ARF6 has been implicated in the trafficking of a number of GPCRs [8], [9] and has also been shown to regulate both clathrin-dependent and independent surface cargo binternalization [7]. In human platelets ARF6 is present on platelet membranes and is important for platelet function [10], [11]. Unlike other small G proteins, ARF6 in its active GTP-bound form is readily detectible in resting platelets and upon platelet activation with collagen or convulxin rapidly converts to a GDP-bound form [11]. This decrease in ARF6-GTP levels has been shown to be essential for platelet aggregation, spreading on collagen and activation of the Rho family of GTPases [11].

In the present study, we investigated the regulation of P2Y receptor traffic and function by ARF6 in both cell lines and importantly in human platelets. We find that blockade of ARF6 function blocks P2Y purinoceptor internalization which in turn attenuates receptor resensitization. Furthermore, we demonstrate that ARF6 likely regulates receptor internalization by facilitating dynamin-dependent internalization of these GPCRs.

## Methods

### Materials

Dulbecco’s modified Eagle’s medium (DMEM), Lipofectamine 2000, fetal bovine serum and CellMask™ Deep Red plasma membrane stain were obtained from Invitrogen. Radiochemicals were from Perkin Elmer Life Sciences. Complete protease inhibitor tablets were from Roche. Anti-HA-monoclonal antibody (HA-11), goat anti-mouse fluorescein-conjugated secondary antibody (1∶200) was purchased from Molecular Probes. An Anti-ARF6 mouse monoclonal antibody was obtained from SantaCruz and a rabbit polyclonal anti-ARF1 antibody [12] was provided by Prof. Sylvain Bourgoin (Laval University, Quebec, Canada). SecinH3 was from Ascent Scientific. All other reagents were from Sigma.

### Preparation of Human Platelets

Human blood was drawn from healthy, drug-free volunteers after obtaining their written informed consent on the day of the experiment under ethical approval from the Local Research Ethics Committee, United Bristol Healthcare Trust (Project E5736). Acid citrate dextrose (ACD: 120 mM sodium citrate, 110 mM glucose, 80 mM citric acid, used at 1∶7 vol/vol) was used as anticoagulant. Platelet rich plasma (PRP) was prepared by centrifugation at 200 *g*, for 17 min and platelets were then isolated by centrifugation for 10 min at 1000 *g*, in the presence of 0.02 U/ml apyrase and prostaglandin E_1_ (PGE_1_; 140 nM) for all assays other than measurement of intracellular cyclic AMP (cAMP) where PGE_1_ was omitted. The pellet was resuspended to a density of 4×10^8^ platelets/ml in a modified Tyrodes-HEPES buffer (145 mM NaCl, 2.9 mM KCl, 10 mM HEPES, 1 mM MgCl_2_, 5 mM glucose, pH 7.3). To this platelet suspension 10 µM indomethacin and 0.02 U/ml apyrase were added, and a 30 min resting period was allowed before stimulation.

### Measurement of Cytosolic Free Calcium ([Ca^2+^]_i_) in Platelets

Measurement of cytosolic calcium was performed as previously described [4], [5]. Briefly, 3 µM Fura-2-AM was added to platelet rich plasma, and incubated at 37°C for 45 min in the presence of 10 µM indomethacin. Platelets were centrifuged and re-suspended in modified Tyrodes. ADP (1 nM −10 µM)-induced calcium responses were subsequently measured at 37°C using a Hitachi F-4500 spectrofluorimeter with fluorescence excitation made at 340 nm and 380 nm and emission at 510 nm. The ratio of the emissions (F1/F2) at those wavelengths is directly correlated to the amount of intracellular calcium. Raw data are expressed as F1/F2 whilst collated data is expressed as % Peak calcium response (% 10 µM ADP alone). To induce receptor desensitisation, a desensitising concentration of ADP (10 µM) was added to platelets for 5 min. Subsequently a stimulating concentration of ADP (10 µM) was added, and the response monitored. Since the ADP response in platelets is attenuated following multiple spin wash steps, desensitising ADP was then removed by the addition of 0.2 unit/ml apyrase (10 min), rather than a wash and spin-step, to promote receptor resensitization. Following apyrase treatment a stimulating concentration of ADP (10 µM) was again added, and the response measured. In all experiments, non-desensitised controls were performed where no desensitising ADP was added. Similarly, the responses of (non-desensitised) platelets to ADP were determined in the presence and absence of apyrase in order to verify that apyrase did not affect stimulations.

### Measurement of cAMP Levels in Platelets

P2Y_12_ purinoceptor activity was assessed in human platelets as previously described [5]. Briefly, platelets were stimulated in the presence of the phosphodiesterase inhibitor IBMX (100 µM) ± forskolin (1 µM) in the absence or presence of ADP (10 µM) for 5 min at 37°C. Cyclic AMP accumulation was terminated by addition of ice cold 100% trichloroacetic acid (TCA) and samples were left to lyse on ice for 1–2 hrs. The resulting samples were spun at 4000 *g* for 5 min and the cAMP-containing supernatant neutralized with 1 M NaOH and TE buffer (50 mM Tris-HCl, 4 mM EDTA, pH 7.4). Cyclic AMP levels were subsequently determined in each sample using a binding assay as previously described [5]. Receptor desensitization was performed as previously described [5]. Briefly, in order to induce receptor desensitization platelets were stimulated with ADP (10 µM; 5 min). In order to induce receptor resensitization apyrase (0.2 unit/ml; 10 min) was subsequently added to remove desensitizing ADP from platelets. As previously described 1 mM EGTA was added 1 min prior to cAMP accumulation experiments to negate calcium dependent-apyrase activity [5]. cAMP accumulation assays were performed on non-desensitised control, desensitised or resensitized platelets. Data are presented as % inhibition of forskolin-stimulated adenylyl cyclase.

### Radioligand Binding in Human Platelets

Platelets were pre-treated with SecinH3 (15 µM; 15 min) or vehicle alone. To induce receptor internalization platelets were subsequently stimulated with ADP (10 µM, 5 min) or vehicle alone. To induce subsequent receptor recycling ADP was removed with apyrase (0.2 unit/ml; 10 min). P2Y_1_ and P2Y_12_ surface receptor expression was subsequently determined by ligand binding in fixed platelets as previously described [4].

**Figure 1:**
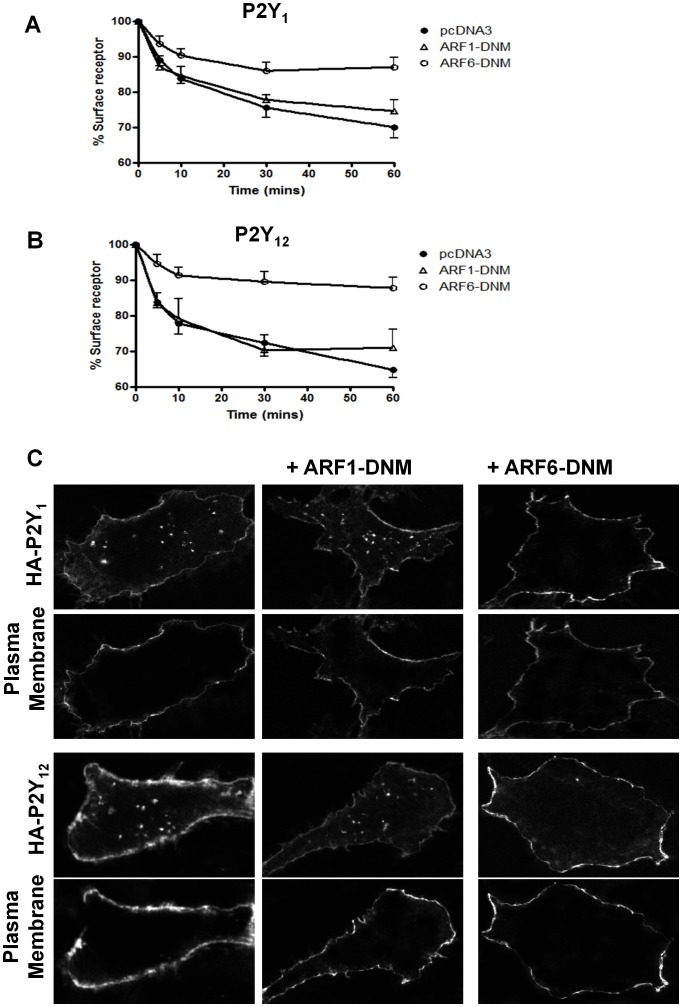
Over-expression of ARF6-DNM attenuates agonist-internalization of both the P2Y_1_ and P2Y_12_ purinoceptor. 1321N1 human astrocytoma cells stably expressing either HA-tagged P2Y_1_ and P2Y_12_ purinoceptor were transiently transfected with either constitutively inactive mutant forms of ARF6 (ARF6 T27N; ARF6-DNM) or ARF1 (ARF1 T31N; ARF1-DNM). Over-expression of DNM forms of ARF1 or ARF6 was confirmed by Western Blotting which showed a 4–5 fold increase over endogenous levels of ARF proteins (data not shown). Cells were subsequently treated with ADP (10 µM; 0–30 min) and surface receptor loss assessed by ELISA (Fig. 1A and Fig. 1B) or receptor internalization visualized by confocal immunofluorescent microscopy (Fig. 1C). In Figure 1A and 1B the data represent means ± SEM of five independent experiments. *p<0.05 compared with respective vector alone controls (Mann–Whitney *U*-test). In Figure 1C receptor cell surface expression was assessed by examining co-localization with a plasma membrane marker (CellMask™ Plasma Membrane Stain).

### Measurement of Platelet Aggregation

Washed platelets were pre-treated with SecinH3 (15 µM; 15 min) or vehicle alone. Platelets were stimulated with ADP (10 µM) in the presence of 1 mg/mL fibrinogen and platelet aggregation measured in a Born optical aggregometer (Chrono-log, Havertown, PA, USA) at 37°C under continuous stirring at 800 g.

### Cell Culture

1321N1 Human astrocytoma cells stably expressing either hemagglutinin (HA)-Tagged human P2Y_1_ or P2Y_12_ receptor were Generated as previously described [5]. cells were maintained in dmem supplemented with 10% fetal bovine serum, 100 units ml^−1^ penicillin G, 100 µG ml^−1^ streptomycin sulfate and 400 µG/ML geneticin at 37°C supplemented with in a humidified atmosphere of 95% air, 5% CO_2_.

**Figure 2:**
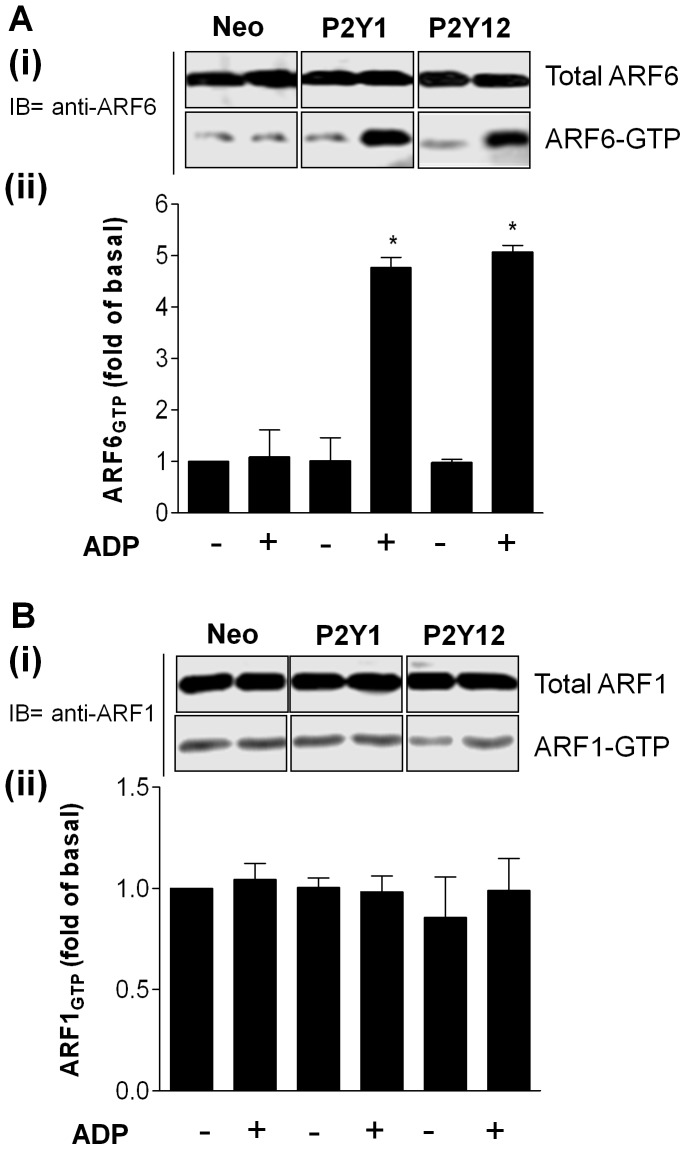
P2Y_1_ and P2Y_12_ purinoceptor stimulation increases ARF6-GTP levels in 1321N1 cells. 1321N1 human astrocytoma cells stably expressing either HA-tagged P2Y_1_ or P2Y_12_ purinoceptor were treated with ADP (10 µM; 5 min) and analysed the endogenous ARF-GTP levels by using GST-GGA3 VHS-GAT pull down assay. Total ARF6 (**A**) or ARF1 (**B**) expression in the cell lysates and ARF6-GTP (**A**) or ARF1-GTP (**B**) precipitated with GST-GGA3 VHS-GAT beads were detected by immunoblotting using an anti-ARF6 mouse monoclonal and an anti-ARF1 rabbit polyclonal antibodies. The relative intensity of each ARF6-GTP or ARF1-GTP band was normalised to total ARF6 or ARF1 measured by densitometry. Values are mean ± SEM from five separate experiments. *p<0.05 compared with no ADP treatment alone control (Mann–Whitney *U*-test).

### ARF6 Activation Assay in 1321N1 Cells and Human Platelets

ARF6 activation was assessed by using the GST pulldown assay as described previously [13], [14]. Briefly, 1321N1 human astrocytoma cells stably expressing either HA-tagged human P2Y_1_ or P2Y_12_ receptor were grown in 10-cm plates were washed twice with ice-cold PBS and harvested using 0.5 ml of ice-cold lysis buffer (50 mM Tris-HCl, pH 7.5, 150 mM NaCl, 1% Triton X-100, 0.5% sodium deoxycholate, 0.1% SDS and 10 mM MgCl_2_) with 1% protease inhibitors mix (Sigma). The cell lysates were incubated with glutathione-Sepharose beads coupled to 50 µg of purified GST-GGA3 VHS-GAT fusion protein at 4°C for 2 hours. The beads were washed three times with the wash buffer (50 mM Tris-HCl, pH 7.5, 10 mM MgCl_2_, 150 mM NaCl and 1% Triton X-100). The lysates that not incubated with the beads were used as an input controls. ARF6-GTP or ARF1-GTP bound to the beads and total ARF6 and ARF1 in the inputs were determined by immunoblotting using either an anti-ARF6 mouse monoclonal antibody or an anti-ARF1 rabbit polyclonal antibody [15]. Immunoblots were scanned and the GTP-bound ARF6 or ARF1 precipitated with GST-GGA3 VHS-GAT beads was normalised to total ARF6 or ARF1 levels in the lysates. GST-GGA3 VHS-GAT fusion protein was expressed in BL21(DE3) strain of *E. coli* and coupled to glutathione-beads as described [16].

**Figure 3:**
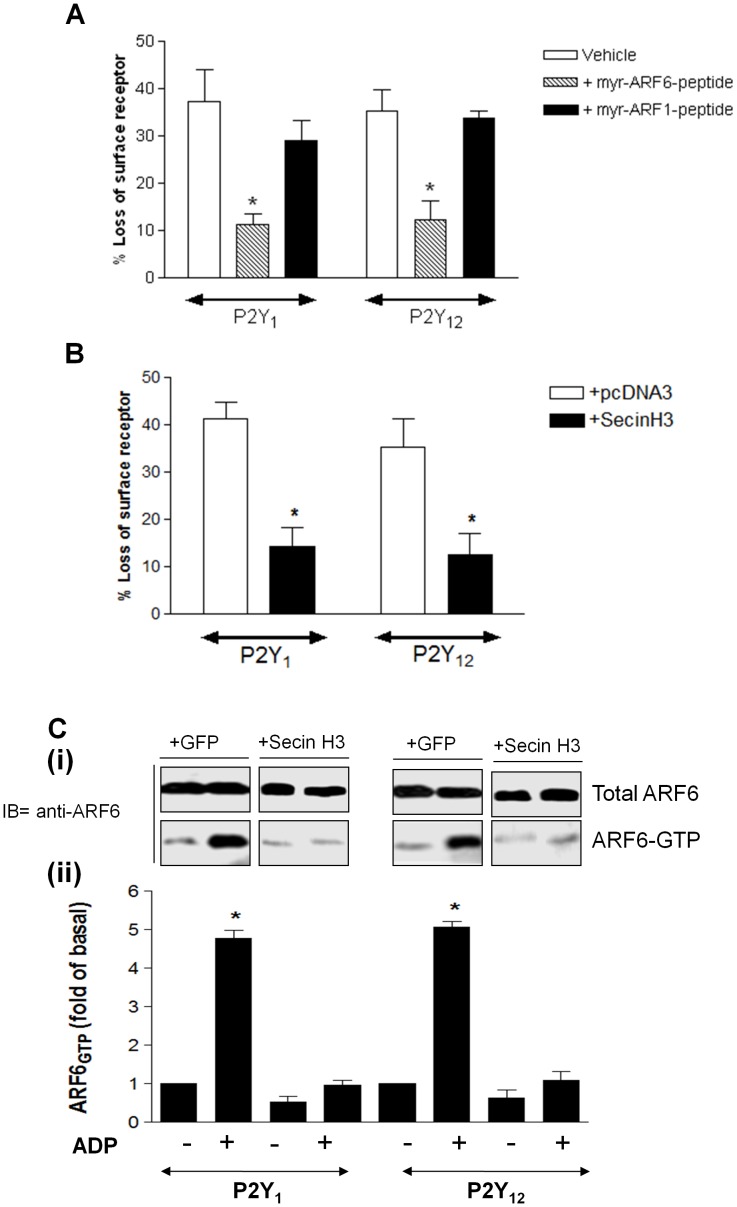
Inhibition of ARF6 activity in 1321N1 cells attenuates internalization of both the P2Y_1_ and P2Y_12_ purinoceptor. 1321N1 human astrocytoma cells stably expressing either HA-tagged P2Y_1_ and P2Y_12_ purinoceptor were incubated with either (**A**) penetratin-coupled-myristoylated (Myr) inhibitory ARF peptides (myr-ARF1-peptide and myr ARF6-peptide) or penetratin vehicle alone (Vehicle) or (B and C) SecinH3 (15 µM) or vehicle control for 30 minutes prior to study. In (**A**) and (**B**) cells were subsequently treated with ADP (10 µM; 30 min) and surface receptor loss assessed by ELISA. The data represent means ± SEM of five independent experiments. *p<0.05 compared with respective control (Mann–Whitney *U*-test). In (**C**) inhibition of P2Y-stimulated ARF6-GTP levels by SecinH3 treatment was assessed. Cells expressing either HA-tagged P2Y_1_ and P2Y_12_ purinoceptor were pretreated with SecinH3 (15 µM) or vehicle control for 30 minutes prior to study. Total ARF6 expression in the cell lysates and ARF6-GTP precipitated with GST-GGA3 VHS-GAT beads were detected by immunoblotting using an anti-ARF6 mouse monoclonal. The relative intensity of each ARF6-GTP band was normalised to total ARF6 and measured by densitometry. Values are mean ± SEM from three separate experiments. *p<0.05 compared with no ADP treatment alone control (Mann–Whitney *U*-test).

### Internalization and Immunofluorescence Microscopy of HA-P2Y_1_ and HA-P2Y_12_ in 1321N1cells

HA-tagged surface receptor loss was assessed by ELISA as described previously [17]. Briefly, cells plated at a density of around 6×10^5^ cells per 60 mm dish were transiently transfected with pcDNA3 containing dominant-negative mutant (DNM) of ARF6 (ARF6 T27N; ARF6-DNM), or ARF1 (ARF1 T31N; ARF1-DNM) or Nm23-H1 (H118C Nm23-H1, a kinase defective) [9], [18]. Twenty-four hours post-transfection, cells were split into 24-well tissue culture dishes coated with 0.1 mg ml^−1^ poly-L-lysine. Twenty-four hours later, cells were incubated with DMEM containing apyrase (0.1 unit/ml; 1 h) and SecinH3 (15 µM; 30 min) or vehicle alone. In parallel experiments, cells were incubated for 30 minutes with penetratin-coupled-myristoylated (Myr) inhibitory ARF peptides (myr-ARF1-peptide and myr ARF6-peptide) or penetratin vehicle alone (Vehicle). Cells were then washed and challenged with DMEM containing ADP (10 µM) for 0–15 min at 37°C. In order to induce receptor recycling apyrase was added (0.2 unit/ml) to remove ADP. Changes in surface receptor expression were subsequently determined by an immunosorbent assay (ELISA) taking advantage of the HA-epitope tag [17], and expressed as either % surface receptor or % loss of surface receptor with the background signal from pcDNA3-transfected controls subtracted from all receptor-transfected values.

**Figure 4:**
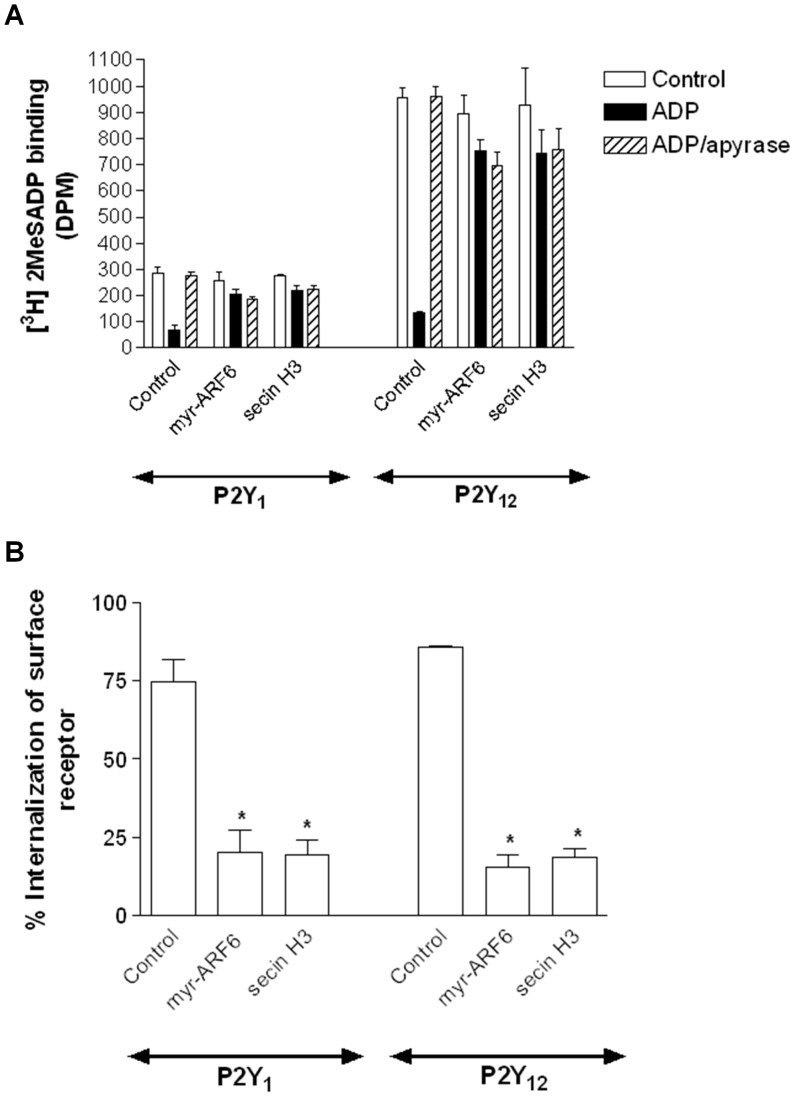
P2Y_1_ and P2Y_12_ purinoceptor internalization is ARF6-dependent in human platelets. Platelets were incubated with either penetratin-coupled-myristoylated (Myr) inhibitory ARF6 peptide or penetratin vehicle alone or SecinH3 (15 µM) for 30 minutes prior to study. Platelets were subsequently exposed to ADP (10 µM; 5 min) to promote receptor internalization. Surface receptor levels were subsequently measured in fixed platelets using [^3^H]-2MeSADP (100 nM) in the presence of either the P2Y_1_ receptor antagonist A3P5P (1 mm) or the P2Y_12_ receptor antagonist AR-C69931MX (1 µm). In (**A**) data are expressed as [^3^H]-2MeSADP (DPM) and in (**B**) data are expressed as percent internalization of surface receptor. Values are mean ± SEM from four separate experiments. In (**B**) *p<0.05 compared with respective vehicle control (Mann–Whitney *U*-test).

**Figure 5:**
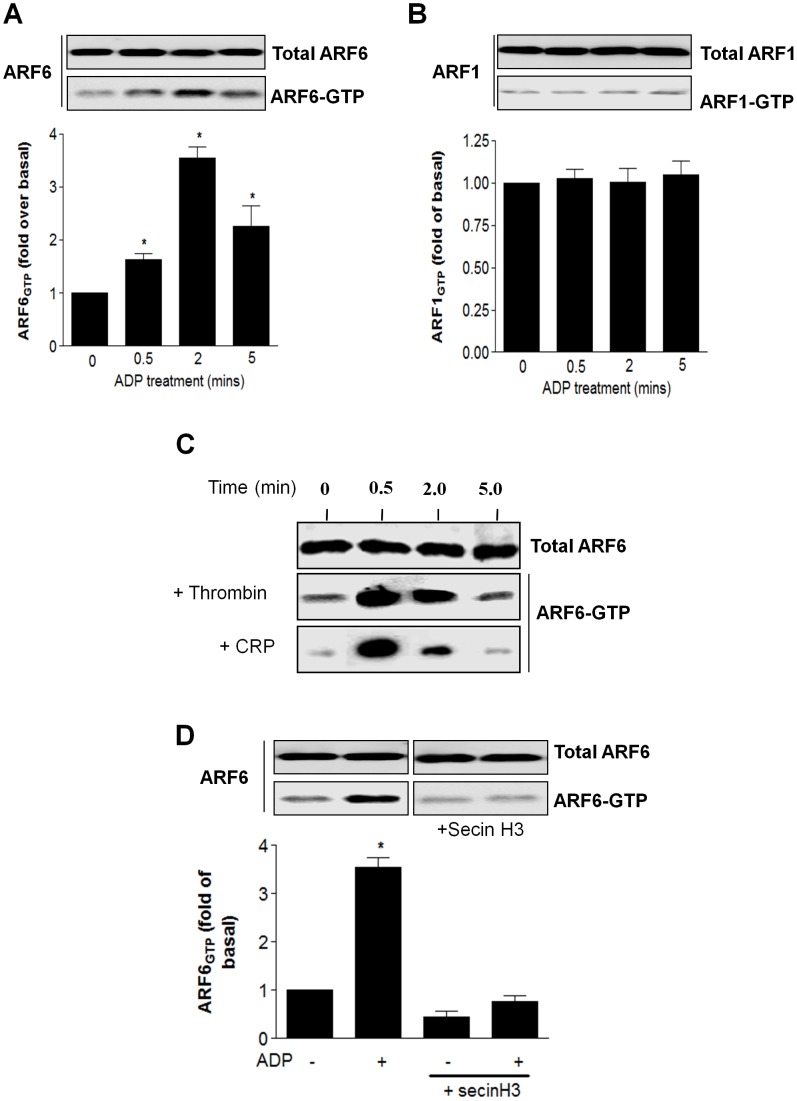
P2Y purinoceptor stimulation increases ARF6-GTP levels in human platelets. (**A** and **B**) Human platelets were treated with ADP (10 µM; 0–5 min) and endogenous ARF-GTP levels assessed by using GST-GGA3 VHS-GAT pull down assay. Total ARF6 (**A**) or ARF1 (B) expression in platelet cell lysates and ARF6-GTP (A) or ARF1-GTP (B) precipitated with GST-GGA3 VHS-GAT beads were detected by immunoblotting using an anti-ARF6 mouse monoclonal and an anti-ARF1 rabbit polyclonal antibodies. The relative intensity of each ARF6-GTP or ARF1-GTP band was normalised to total ARF6 or ARF1 measured by densitometry (**A** and **B**). Values are mean ± SEM from three separate experiments. *p<0.05 compared with no ADP treatment alone control (Mann–Whitney *U*-test). (**C**) Human platelets were treated with thrombin (0.3 unit/ml) and collagen-related peptide (CRP; 5 µg/ml) for 0–5 mins and and endogenous ARF-GTP levels assessed. The data are representative of three independent experiments (**D**) Platelets were incubated with SecinH3 (15 µM) or vehicle alone for 30 minutes. Platelets were then treated with ADP (10 µM; 0–5 min) and endogenous ARF6-GTP levels assessed. The data represent mean ± SEM of three independent experiments. *p<0.05 compared with no ADP treatment alone control (Mann–Whitney *U*-test).

Cellular distribution of HA-tagged receptor in 1321N1 cells was assessed by immunofluorescence microscopy [17]. Briefly cells were grown on poly-L-lysine coated coverslips in 6 well plates. Twenty-four hours later, receptor distribution was assessed using a primary anti-HA-monoclonal antibody (HA-11; 1∶200) and goat anti-mouse fluorescein-conjugated secondary antibody (1∶200). Cell plasma membrane was stained using CellMask™ Deep Red plasma membrane stain. Coverslips were mounted using Slow-Fade mounting medium and examined by microscopy on an upright Leica TCS-NT confocal laser scanning microscope attached to a Leica DM IRBE epifluorescence microscope with phase-contrast and a Plan-Apo 40×1.40 NA oil immersion objective. All images were collected on Leica TCS-NT software for 2D and 3D image analysis and processed using Adobe Photoshop 6.0.

**Figure 6:**
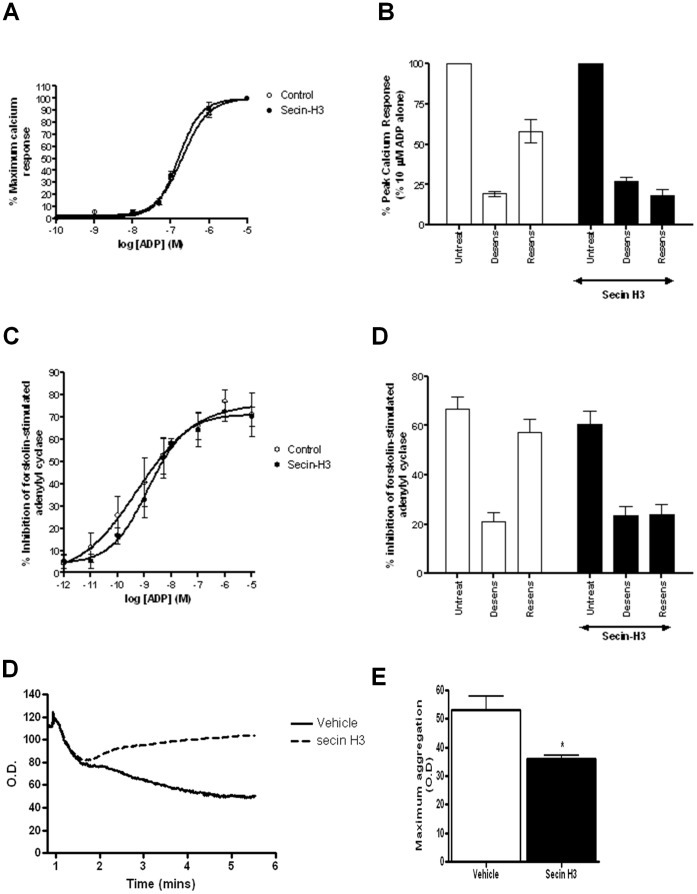
ARF6-dependent P2Y_1_ and P2Y_12_ purinoceptor internalization is required for effective receptor regulation in human platelets. Platelets were incubated with SecinH3 (15 µM) or vehicle alone for 30 minutes and P2Y_1_ (**A** and **B**) or P2Y_12_ (**C** and **D**) purinoceptor activity subsequently assessed. In (**A**) and (**B**) P2Y_1_ purinoceptor stimulated calcium signalling was assessed. (**A**) Full dose response curve of ADP (1 nM-10 µM)-stimulated of P2Y_1_ purinoceptor activity. (**B**) P2Y_1_ purinoceptor desensitization was assessed by comparing receptor activity (ADP; 10 µM) before (untreat) and after pretreatment with ADP (10 µM; 5 min; desens). Subsequent receptor resensitization (resens) was assessed following removal of desensitizing ADP with apyrase (0.2 unit mL^−1^; 10 min). Data are expressed as the percent peak calcium response obtained from the initial control ADP (10 µM) response. The data represent mean ± SEM of four independent experiments. p<0.05 compared with untreated control (Mann–Whitney *U-*test). #p<0.05 compared with resensitized control in the absence of SecinH3 treatment (Mann–Whitney *U*-test). In (**C** and **D**) P2Y_12_ purinoceptor stimulated inhibition of forskolin (1 µm; 5 min)-stimulated adenylyl cyclase activity was assessed. (**C**) Full dose response curve of ADP (0.01 nM-10 µM)-stimulated P2Y_12_ purinoceptor activity. (**D**) P2Y_12_ purinoceptor desensitization was assessed by comparing receptor activity (ADP; 10 µM) before (untreat) and after pretreatment with ADP (10 µM; 5 min; desens). Subsequent receptor resensitization (resens) was assessed following removal of desensitizing ADP with apyrase (0.2 unit mL^−1^; 10 min). Data are expressed as the percentage inhibition of forskolin-stimulated adenylyl cyclase. The data represent mean ± SEM of four independent experiments. *p<0.05 compared with untreated control (Mann–Whitney *U*-test). #p<0.05 compared with resensitized control in the absence of SecinH3 treatment (Mann–Whitney *U*-test). In (**E** and **F**) Washed platelets were pretreated with SecinH3 (100 µM; 15 min) in the presence of 1 mg/ml fibrinogen. Platelets were subsequently stimulated with ADP (10 µM), and the resultant aggregation response was monitored by optical aggregometry. (**E**) Shows a representative trace from at least three experiments with ADP added at time point 0 and aggregation expressed as optical density (O.D.) In (**F**) data are expressed as maximum aggregation (O.D.) following 300 seconds ADP stimulation. The data represent mean ± SEM of three independent experiments. *p<0.05 compared with untreated control (Mann–Whitney *U*-test).

**Figure 7:**
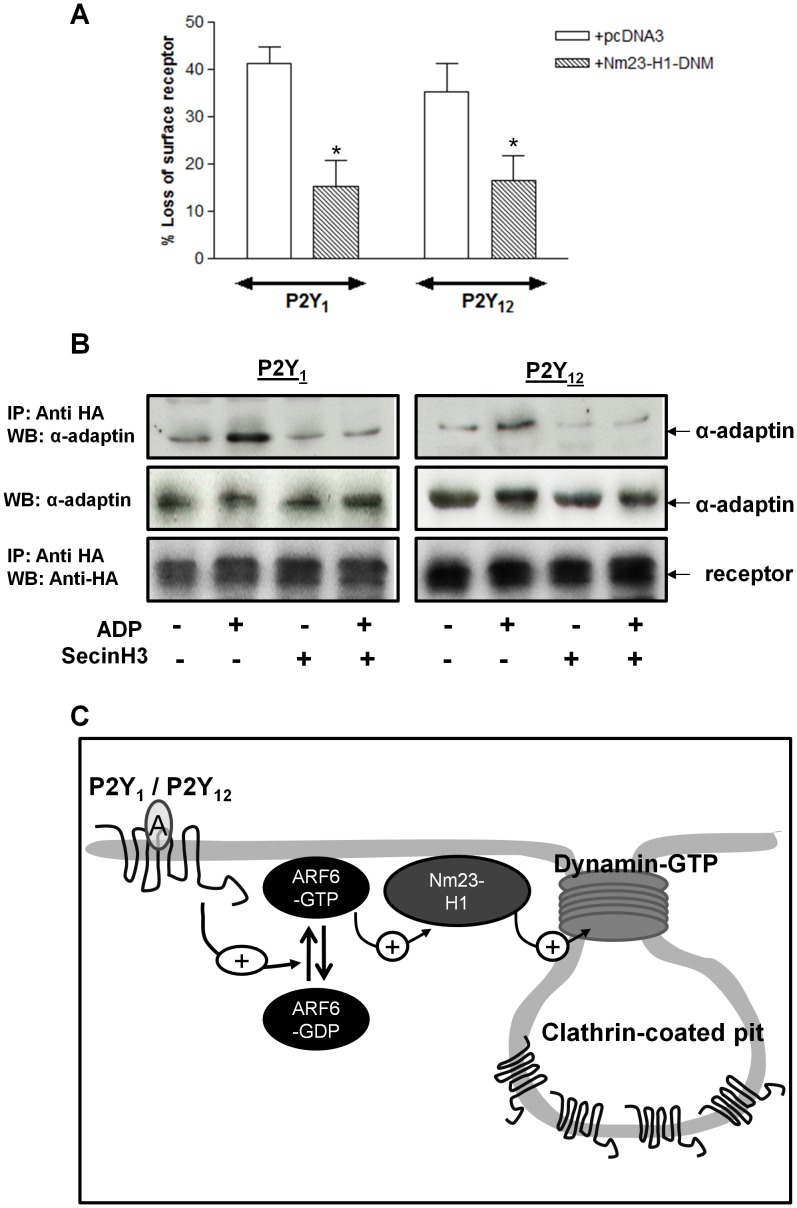
ARF6 regulates aspects of clathrin- and dynamin-dependent P2Y_1_ or P2Y_12_ purinoceptor receptor internalization. (**A**) 1321N1 human astrocytoma cells stably expressing either HA-tagged P2Y_1_ and P2Y_12_ purinoceptor were transfected with a dominant negative mutant of the Nucleoside diphosphate kinase Nm23-H1 (H118C Nm23-H1). Cells were subsequently treated with ADP (10 µM; 30 min) and surface receptor loss assessed by ELISA. The data represent mean ± SEM of four independent experiments. *p<0.05 compared with pcDNA3 vector control (Mann–Whitney *U*-test). (**B**) 1321N1 human astrocytoma cells stably expressing either HA-tagged P2Y_1_ and P2Y_12_ purinoceptor were treated with SecinH3 (15 µM) for 30 minutes. Cells were subsequently stimulated with ADP (10 µm; 5 min) at 37°C. Reactions were stopped by addition of ice-cold lysis buffer and receptor was immunoprecipitated from cell lysates using an anti-HA antibody (HA-11) and association with endogenous α-adaptin assessed by immunoblotting. Whole-cell lysates (WCL) lanes are included as positive controls for detection by anti-α-adaptin antibodies as are lanes showing equal levels of receptor immunoprecipitation. (**C**) Model of ARF6-dependent internalization of P2Y_1_ and P2Y_12_ purinoceptor. Activation of the P2Y_1_ and P2Y_12_ purinoceptor increases ARF6 activation. ARF6-GTP in turn stimulates Nm23-H1 which in turn promotes dynamin-dependent internalization of the P2Y_1_ and P2Y_12_ purinoceptors.

### Experimental Design and Statistics

Data were analysed by the iterative fitting program GraphPAD Prism (GraphPAD Software). Log concentration-effect curves were fitted to logistic expressions for single-site analysis, whilst t0.5 values for agonist-induced internalization were obtained by fitting data to single exponential curves. Where appropriate statistical significance was assessed by Mann-Whitney-U test or by two-way ANOVA.

## Results

In order to study the role of ARF6 in either P2Y_1_ or P2Y_12_ purinoceptor function and circumvent the inherent methodological problems that this presents in platelets, we initially examined these receptors in a cell line system. In these studies we used 1321N1 human astrocytoma cells stably expressing N-terminal HA-epitope tagged versions of either receptor. Using these cells we are able to quantify receptor signaling and agonist-induced surface receptor loss by ELISA and immunofluorescence microscopy as previously described [17]. We initially sought to determine the effects of ARF6 on agonist-induced P2Y_1_ and P2Y_12_ purinoceptor internalization. Cells were transiently transfected with a constitutively inactive mutant form of ARF6 (ARF6 T27N; ARF6-DNM), previously shown to inhibit ARF6-dependent internalization of the β_2_-adrenoceptor [9]. Expression of this construct significantly attenuated both P2Y_1_ (Fig. 1A) and P2Y_12_ (Fig. 1B) purinoceptor as assessed by ELISA (Fig. 1A and 1B). Expression of a constitutively inactive form of ARF1 (ARF1 T31N; ARF1-DNM) had no effect on either P2Y_1_ (Fig. 1A) or P2Y_12_ (Fig. 1B) purinoceptor internalization. Western blotting confirmed that both ARF1-DNM and ARF6-DNM are expressed after transient transfection (70–90% cells transfected at 4–5 fold over endogenous ARF6 levels; data not shown). Confocal imaging of P2Y_1_ and P2Y_12_ purinoceptor localization showed that whereas in control cells or those transfected with ARF1-DNM, agonist treatment led to the marked accumulation of receptor in endosome-like structures as previously described in the cell cytoplasm, this accumulation was greatly reduced when ARF6-DNM was cotransfected into the cells. Comparison with a plasma membrane dye suggested that both the P2Y_1_ and P2Y_12_ purinoceptor was retained at the cell surface following ARF6-DNM expression.

Given these findings we next sought to determine if activation of either of these receptors may increase ARF6 activity (Fig. 2). Active ARF-GTP binds specifically to VHS-GAT domain of GGA3, a downstream effector. Therefore, the VHS-GAT of GGA3 can be used as a probe to specifically isolate the active forms of ARF1 and ARF6. We therefore examined ARF6 activation using a GST–GGA3 pull down assay as previously described [13], [14], [16]. P2Y_1_ or P2Y_12_ purinoceptor expressing cells were transiently transfected with HA-tagged forms of ARF1 or ARF6. Importantly ADP-treatment of these cells specifically increased ARF6-GTP levels in these cells whilst ARF1-GTP levels were unaffected.

We subsequently used two alternative strategies to inhibit endogenous ARF6 activity in these cells, using either a peptide or chemical inhibitor approach (Fig. 3). Given the anuclear nature of platelets we intended to validate these approaches in cell line systems prior to their translation into human platelets. For the peptide strategy two Myristoylated (Myr) inhibitory ARF peptides (myr-ARF1-peptide and myr ARF6-peptide) that contain the N-terminal residues [19]–[20] of these proteins were utilized that are effective in inhibiting the functional activity of ARFs [20]. In order to facilitate entry of these peptides into cells we coupled them to penetratin [21], [22] which has been used successfully to internalize covalently attached peptides and oligonucleotides and to convey them to the cytoplasm and nucleus of many cell types. Cells were therefore incubated with these penetratin-conjugated peptides (myr-ARF1- or myr-ARF6-peptide) or penetratin alone (vehicle) and agonist-induced internalization examined (Fig. 3A). Importantly as with our DNM studies treatment with only the myr-ARF6-peptide inhibited ADP-stimulated receptor internalization as assessed by ELISA.

For the chemical approach we used SecinH3, a Sec7-specific Guanine nucleotide exchange factor (GEF) inhibitor that displays selectivity for GEFs of the cytohesin family [23], [24]. It is well established that the binding of the ARF6-GEF cytohesin 2 (also known as ARNO) promotes the exchange of GDP for GTP promoting ARF6 activation. Therefore inhibition of ARNO with SecinH3 will inhibit ARF6 activation. We have recently demonstrated that SecinH3 inhibits the agonist-induced internalization of Luteinizing Hormone Chorionic Gonadotropin (LHCG) receptor [18]. Treatment with SecinH3 also blocked agonist-stimulated P2Y_1_ or P2Y_12_ purinoceptor internalization (Fig. 3B). Importantly treatment of 1321N1 cells with SecinH3 inhibited ADP-stimulated ARF6-GTP-levels in either P2Y_1_ or P2Y_12_ purinoceptor expressing cells (Fig. 3C). Given these results with the Myr-ARF6-peptide and SecinH3 in cell lines we next examined the ability of these agents to inhibit P2Y purinoceptor internalization in human platelets.

We have previously used a ligand binding approach to examine P2Y_1_ or P2Y_12_ purinoceptor internalization and recycling in human platelets [17]. We examined surface expression of ADP receptors on platelets by ligand binding using [^3^H]-2MeSADP (100 nM) in the presence of A3P5P (1 mM) or AR-C69931MX (1 µM) to distinguish either the P2Y_1_ or P2Y_12_ surface binding sites as previously reported [4], [17]. Importantly treatment with Myr-ARF6 peptide or SecinH3 did not significantly change control/basal levels or either P2Y_1_ or P2Y_12_ purinoceptor surface expression (clear bars Fig. 4A). As in our previous studies, following pretreatment with ADP (10 µm; 5 min), its subsequent removal with apyrase (0.2 unit mL^−1^; 3 min) and platelet fixation, there was a clear reduction in [^3^H]-2MeSADP binding to both P2Y_1_ and P2Y_12_ compared with apyrase-alone treated controls. Data shown are summarized in Figure 4B. As expected, if we lengthened the period of apyrase exposure to 10 minutes (ADP/apyrase), we found that both the P2Y_1_ and P2Y_12_ surface receptor levels returned to control levels (Fig. 4A). We next examined if receptor internalization was impaired in platelets treated with Myr-ARF6 peptide or SecinH3 (Fig. 4A and 4B). Importantly pretreatment with either Myr-ARF6-peptide or SecinH3 inhibited ADP stimulated receptor internalization (Fig. 4A and 4B).

We next sort to determine if either P2Y_1_ or P2Y_12_ purinoceptor activation in human platelets promoted ARF6 activation. We again made use of the ability of active ARF-GTP to bind specifically to VHS-GAT domain of GGA3, a downstream effector as previously described [13], [14], [16]. Importantly activation of human platelets with ADP (10 µM) promoted a transient but robust increase in ARF6-GTP levels (Fig. 5A) whilst ARF1-GTP levels (Fig. 5B) were unaffected. Given this difference to other studies where ADP treatment was shown to have no effect on ARF6-GTP levels [10], [11], we further examined if other platelet agonists could increase ARF6-GTP levels. Interestingly we found that aswell as ADP addition of both thrombin (0.3 unit/ml) and collagen-related peptide (CRP; 5 µg/ml) could promote a rapid and transient increase in ARF6-GTP levels (Fig. 5C). Importantly pretreatment of platelets with SecinH3 attenuated ADP-dependent increases in ARF6-GTP levels (Fig. 5D) indicating that in human platelets SecinH3 was effectively inhibiting ARF6 activation.

Therefore we next examined if treatment with SecinH3 attenuated receptor P2Y_1_ or P2Y_12_ purinoceptor signaling desensitization or resensitization. P2Y_1_–stimulated rises in intracellular calcium and P2Y_12_-inhibition of forskolin-stimulated adenylyl cyclase were measured as is standard in our laboratory [5]. SecinH3 pretreatment did not significantly attenuate the acute ADP-dependent signaling of either the P2Y_1_ or P2Y_12_ purinoceptor (Fig. 6A and 6C). In order to examine receptor desensitization and resensitization platelets were pretreated with ADP to desensitize receptor responses, and then allowed to recover in the presence of apyrase added to remove desensitizing ADP to examine resensitization of responses as previously described [4]. As expected and previously reported [4], pretreatment with agonist (ADP; 10 µM; 5 min) decreased subsequent ADP-stimulated P2Y_1_ (Fig. 6B) and P2Y_12_ (Fig. 6D) purinoceptor responses whilst prolonged addition of apyrase (0.2 units/ml; 30 mins) led to a resensitization of both receptor responses. Pretreatment with SecinH3 did not significantly affect receptor desensitization but did completely abolish receptor resensitization (Figure 6B and 6D). Therefore ARF6-dependent internalization of the P2Y_1_ or P2Y_12_ purinoceptor is required for effective resensitization of these receptors. We subsequently tested if ARF6 also played any role in regulating ADP-dependent platelet aggregation. Importantly we found that pretreatment with SecinH3 (100 µM; 15 mins) did significantly attenuate ADP (10 µM)-dependent platelet aggregation (sample trace Figure 6E with averaged data Figure 6F).

Given these results and previous studies from our group we next sought to examine how ARF6 may regulate P2Y_1_ or P2Y_12_ purinoceptor internalization. Both of these GPCRs internalize in a clathrin- and dynamin-dependent manner in human platelets. ARF6 has been shown to regulate the activity of dynamin by an interaction with the Nucleoside diphosphate kinase Nm23-H1 [25], [26]. ARF6-interacts with and recruits Nm23-H1 to the cell membrane where it provides a local source of GTP for the GTPase dynamin. Unfortunately given the lack of an applicable approach (either chemical or peptide) to examine the function of Nm23-H1 in human platelets we examined the ability of this protein to regulate P2Y_1_ or P2Y_12_ purinoceptor internalization in 1321N1 cells. In these studies we used a DNM of Nm23-H1 (H118C Nm23-H1; [26]) that we have recently shown to inhibit the ARF6-dependent internalization of another GPCR, the LHCG receptor [18]. Expression of this DNM did indeed significantly attenuate both P2Y_1_ and P2Y_12_ purinoceptor internalization (Figure 7A).

ARF6 activation has been shown to increase GPCR association to components of clathrin-coated pits (CCPs) [27]. Therefore we examined if this was also the case for the P2Y_1_ or P2Y_12_ purinoceptor (Fig. 7B). We have previously shown that these receptors associate with α-adaptin, a core component in CCPs following ADP stimulation [17]. We therefore examined if receptor/α-adaptin association was reduced following pre-treatment with SecinH3. As expected ADP stimulation (10 µM; 5 min) increased P2Y_1_ and P2Y_12_ purinoceptor/α-adaptin association (Fig. 7B). Importantly SecinH3 pretreatment significantly attenuated this association. Therefore we postulate that ARF6 promotes clathrin-dependent internalization of these receptors by increasing receptor association with CCP components and through the recruitment of Nm23-H1 (Fig. 7C).

## Discussion

In the present study, we describe the novel finding that the small GTPase ARF6 regulates the clathrin-dependent internalisation of both P2Y_1_ and P2Y_12_ purinoceptors and is required for the maintenance of receptor function in human platelets. The activation of P2Y_1_ and P2Y_12_ purinoceptors by ADP is critical for normal platelet function, performing a pivotal role in the formation of stable platelet aggregates with continuous ADP signaling facilitating thrombus stability [28], [29]. Previous studies from our laboratory have shown that the responses of P2Y_1_ and P2Y_12_ are able to rapidly respond to changes in circulating ADP levels and that the sensitivity of these receptors to agonist is continuously regulated in order to avoid inappropriate thrombosis. Receptor desensitization and then subsequent resensitization represent key mechanisms by which the delicate balance between rest and activation of platelets in the circulation is maintained.

Upon prolonged exposure to ADP, the responsiveness of both P2Y_1_ and P2Y_12_ purinoceptors desensitize via different protein kinase C (PKC)- and GRK-dependent mechanisms, respectively [5], and subsequently enter distinct subpopulations of CCPs prior to internalisation in human platelets [17]. More recently, we have demonstrated that following clathrin-dependent agonist-induced internalisation, receptor dephosphorylation and subsequent receptor recycling is required for the rapid resensitisation of P2Y purinoceptor function [4].

Although we have previously demonstrated that P2Y purinoceptors internalize in human platelets in a dynamin-dependent manner little is still known about the molecular mechanisms underlying this process in human platelets. The small GTPase ARF6, which is endogenously expressed in human platelets [10] regulates both clathrin-dependent and independent endocytic pathways of multiple membrane cargo including many GPCRs [8], [9]. Our initial studies in cell lines demonstrated that blockade of ARF6 activity, either by the use of ARF6-DNM, Myr-ARF6-peptide or ARF6-GEF chemical inhibitor SecinH3, attenuated P2Y_1_ and P2Y_12_ purinoceptor internalization. Further in our cell line studies we demonstrated that activation of these receptors promoted ARF6 activation. Therefore by their activation these receptors are able to increase ARF6 activity and promote their own internalization. Further detailed study will be required to determine how receptor stimulation increases ARF6 activity although it should be noted that the P2Y_12_ purinoceptor associates with arrestins upon receptor activation [17]. Arrestins have been shown to associate with ARF6 and potentiate its activation by the ARF6-GEF ARNO, which has also been found in complex with arrestins [30].

Classically, ARF proteins are known as molecular switches, by cycling between active GTP-bound and inactive GDP-bound conformations, that coordinate several cellular events such as vesicle trafficking, actin remodeling, and modification of membrane lipid content. For example, at the Golgi, the activation of ARF1 can promote the recruitment of COP I, COP II, and clathrin; components necessary for the formation of trafficking vesicles [31], [32], [33]. At the plasma membrane, ARF6 is able to promote clathrin/AP-2 recruitment to synaptic membranes by activating the phosphatidylinositol phosphate kinase type 1γ [34]. The ability of ARF6 to regulate receptor internalization has now been demonstrated for a number of GPCRs including the β_2_-adrenergic (β_2_AR) [8] and TP thromboxane receptors [35]. Indeed, depletion of ARF6 by siRNA results in a marked inhibition of receptor internalization utilizing different endocytic pathways demonstrating the importance of this small GTP-binding protein in regulating the endocytic process [8]. Interestingly for the angiotensin II AT1a receptor ARF6 coordinates the recruitment of AP-2 and clathrin, integral proteins found in CCPs to activated receptors during the endocytic process promoting receptor internalization [27]. Both the P2Y_1_ and P2Y_12_ purinoceptor internalize in a clathrin-dependent manner. Our studies show that blockade of ARF6 activity attenuates P2Y_1_ and P2Y_12_ purinoceptor association with α-adaptin, a core component of CCPs.

We also find that Nm23-H1, an NDP-kinase, regulates P2Y purinoceptor internalization. Nm23-H1 is encoded by the *Drosophila melanogaster abnormal wing dics (awd)* gene and belongs to a family of structurally conserved NDP-kinase that generate nucleoside triphosphates from respective diphosphates [25], [26]. Nm23-H1 has been shown to provide a source of GTP during dynamin-dependent vesicle fission in nerve terminals [26] and regulate membrane cargo internalization [36] including that of another GPCR the LHCG receptor [18] whilst Nm23-H2 regulates the internalization of GPCRs including the TP thromboxane receptor [37]. ARF6 has been shown to interact with and recruit Nm23-H1 to the cell membrane where it provides a local source of GTP for the GTPase dynamin [26]. We therefore postulate that P2Y purinoceptor activation stimulates ARF6 which in turn promotes clathrin-dependent receptor internalization via an Nm23-H1-dependent stimulation of dynamin activity.

Importantly in our studies we have translated our findings with over-expressed receptors in cell lines into human platelets. Both the Myr-ARF6-peptide and SecinH3 block P2Y_1_ and P2Y_12_ purinoceptors internalization. Importantly although ARF6 blockade does not attenuate acute P2Y purinoceptor signalling it does block receptor resensitization. Importantly we were able to show that ADP-stimulation promoted transient but robust ARF6 activation in platelets. This is different from previous studies where ADP treatment was shown to have no effect on ARF6-GTP levels [10], [11]. There a number of subtle but important differences between these studies and our findings. Previously the full time-course of ADP-dependent increases in ARF6-GTP levels were not investigated whilst platelets were incubated for 2–5 min at 37°C before ADP addition. Importantly, unlike the previous studies, during platelet preparation we incubate our platelets in the presence of the ADP scavenger apyrase (0.02 unit/ml) in order to retain platelet P2Y responsiveness. We find that these receptors are extremely sensitive to ADP secreted during platelet preparation which can desensitize their function [4], [5]. Interestingly we find that without the addition of apyrase we lose platelet ADP-dependent ARF6 activation and even see a transient increase in ARF6 activity on incubating platelets at 37°C which is blocked by the addition of P2Y_1_ and P2Y_12_ receptor antagonists (data not shown). Interestingly we also find a transient increase in both thrombin-activated PAR and collagen-activated GPVI receptors in our studies whereas ARF6-GTP levels have been reported to decrease [10], [11]. The consequences of these changes in ARF6-GTP levels upon P2Y purinoceptor internalization are unclear and require further detailed investigation, although in our study reduced ARF6 function attenuates the internalization and resensitization of these receptors Importantly we also find that inhibition of ARF6 with SecinH3 attenuates ADP-dependent platelet aggregation. We speculate that this attenuation of ADP-dependent platelet aggregation maybe as consequence of reduced P2Y receptor internalization and resensitization although the true physiological consequence of P2Y receptor traffic upon platelet function is still to be determined.

In conclusion this work expands our understanding of the molecular regulators of platelet purinoceptor function. Given the physiological and pathophysiological importance of platelet P2Y purinoceptor function, this study demonstrates the integral importance of the small GTPase ARF6 upon ADP signalling in human platelets. Further study is now required to determine the functional importance of ARF6 upon ADP-dependent thrombus generation and stabilization.
